# The Tunable Parameters of Graphene-Based Biosensors

**DOI:** 10.3390/s24155049

**Published:** 2024-08-04

**Authors:** Talia Tene, Jiří Svozilík, Dennys Colcha, Yesenia Cevallos, Paola Gabriela Vinueza-Naranjo, Cristian Vacacela Gomez, Stefano Bellucci

**Affiliations:** 1 Department of Chemistry, Universidad Técnica Particular de Loja, Loja 110160, Ecuador; 2 Facultad de Ciencias, Escuela Superior Politécnica de Chimborazo (ESPOCH), Riobamba 060155, Ecuador; 3 UNICARIBE Research Center, University of Calabria, 87036 Rende, Italy; 4 College of Engineering, Universidad Nacional de Chimborazo, Riobamba 060108, Ecuador; 5 Universidad San Francisco de Quito IMNE, Diego de Robles s/n, Cumbayá, Quito 170901, Ecuador; 6 INFN-Laboratori Nazionali di Frascati, Via E. Fermi 54, 00044 Frascati, Italy

**Keywords:** surface plasmon resonance, graphene, silver, gold, biosensors, sensitivity of detection, detection accuracy, quality factor, transfer matrix method

## Abstract

Graphene-based surface plasmon resonance (SPR) biosensors have emerged as a promising technology for the highly sensitive and accurate detection of biomolecules. This study presents a comprehensive theoretical analysis of graphene-based SPR biosensors, focusing on configurations with single and bimetallic metallic layers. In this study, we investigated the impact of various metallic substrates, including gold and silver, and the number of graphene layers on key performance metrics: sensitivity of detection, detection accuracy, and quality factor. Our findings reveal that configurations with graphene first supported on gold exhibit superior performance, with sensitivity of detection enhancements up to 30% for ten graphene layers. In contrast, silver-supported configurations, while demonstrating high sensitivity, face challenges in maintaining detection accuracy. Additionally, reducing the thickness of metallic layers by 30% optimizes light coupling and enhances sensor performance. These insights highlight the significant potential of graphene-based SPR biosensors in achieving high sensitivity of detection and reliability, paving the way for their application in diverse biosensing technologies. Our findings pretend to motivate future research focusing on optimizing metallic layer thickness, improving the stability of silver-supported configurations, and experimentally validating the theoretical findings to further advance the development of high-performance SPR biosensors.

## 1. Introduction

Surface plasmon resonance (SPR) biosensors are renowned for their real-time technique and label-free detection capabilities, making them invaluable in biomedical diagnostics, environmental monitoring, and food safety applications [1]. Recent advancements in optical bio-detection methods have significantly enhanced SPR-based biosensing technologies. Integrating plasmonic biosensors with resonant cavity LEDs has achieved an extraordinary optical transmission peak with high sensitivity of detection (for simplicity, sensitivity only, unless otherwise stated), showcasing precise SPR-based bio-detection [2]. Additionally, handheld high-throughput plasmonic biosensors using computational on-chip imaging facilitate multiplexed, label-free detection of biomolecules, relevant to SPR applications [3]. Moreover, integrating optoelectronic tweezers (OET) with spectroscopic analysis allows precise manipulation and spectral characterization of single micro-samples, enhancing SPR-based biosensing [4]. These approaches demonstrate the evolving capabilities of SPR-based optical biosensing technologies.

Due to their excellent plasmonic properties, traditional SPR biosensors typically employ thin films of noble metals such as gold and silver [5]. However, the sensitivity of these sensors can be limited by the choice of substrate and the nature of the metal-dielectric interface. Gold is favored for its chemical stability and resistance to oxidation, which ensures a longer lifespan for the sensor [6]. Additionally, gold provides excellent plasmonic properties that contribute to high sensitivity in detecting refractive index changes. However, gold surfaces exhibit poor interaction with biomolecules [7]. This limitation arises because biomolecules do not adsorb efficiently onto gold, leading to lower detection accuracy and sensitivity in practical applications where biomolecule adsorption is crucial.

Silver, on the other hand, offers even better plasmonic properties than gold, resulting in higher sensitivity [8]. The dielectric constant of silver allows for a sharper and more pronounced SPR signal, which enhances the ability of the sensor to detect minute changes in the refractive index [9]. Despite these advantages, silver is highly susceptible to oxidation, especially when exposed to the aqueous environments typical in biosensing applications [10]. The formation of silver oxide layers on the surface can degrade the performance of the sensor over time, leading to reduced sensitivity and reliability.

In recent years, several works have introduced graphene as a promising material for enhancing the performance of SPR biosensors [11]. Graphene, a two-dimensional carbon allotrope, has unique optical properties, including high electron mobility and a large surface area [12,13], which make it an ideal candidate for improving biosensor sensitivity and detection accuracy [14,15]. The interaction of graphene with biomolecules is significantly stronger than that of gold or silver, primarily due to π-stacking interactions between the hexagonal rings of graphene and carbon-based ring structures in biomolecules [16]. This strong interaction enhances the adsorption efficiency, leading to higher sensitivity and better performance of the biosensor.

In a pioneering work [17], the idea of using graphene as a biomolecular recognition element (BRE) in SPR biosensors was proposed. This concept leverages the high adsorption capacity of graphene for biomolecules, which significantly enhances the sensitivity of the sensor. In particular, the graphene layer serves as a functionalization layer that not only improves the interaction with biomolecules but also modifies the optical properties of the sensor, leading to an increased refractive index change near the sensor surface and, consequently, higher sensitivity (~25%).

With this idea in mind, graphene-based biosensors have become an area of intense research interest, as, remarkably, they were used for detecting biomolecules related to infectious diseases such as SARS-CoV-2 [18,19]. Particularly, the COVID-19 pandemic has pointed out the urgent need for rapid, sensitive, and reliable diagnostic tools. Recent studies also have demonstrated the potential of SPR biosensors based on graphene (or graphene-derived materials), for instance, for detecting serum folate biomarkers [20], dengue virus [21], bacteria [22], glucose [23], anticholera toxin [24], folic acid protein [25], anti-BSA [26], hCG protein [27], CK19 protein [28], anti-PAPP-A2 [29], glycerol [30], among other targets.

As noted, the integration of graphene into SPR biosensors not only improves sensitivity but also expands the range of detectable biomolecules, making these sensors highly versatile for the development of next-generation biosensors. Despite the extensive body of work demonstrating the use of graphene in SPR biosensors and its potential applications [31], it is essential to investigate further specific configurations and parameters that can optimize the performance of graphene-based sensors. 

Such missing research is presented here. Indeed, this study aims to provide a comprehensive theoretical analysis of graphene-based SPR biosensors, focusing on the effects of varying substrates (gold, silver, gold/silver, or silver/gold) and the number of graphene layers on key performance metrics. Using the transfer matrix method (TMM), we analyze how these variables influence sensitivity enhancement, sensitivity to refractive index change, full width at half maximum (FWHM), quality factor, and detection accuracy. 

Our predictions indicated poor performance for the systems with gold/silver and silver/gold. However, by reducing the thickness of each metallic substrate by 30%, we observed significant improvements in sensor properties. This adjustment emphasizes the importance of substrate thickness in optimizing sensor performance. Additionally, by systematically varying these parameters, this research also aims to address previously unexplored aspects and close gaps that are crucial for developing highly sensitive and accurate SPR biosensor prototypes with broad applications in biomedical diagnostics, environmental monitoring, and beyond.

## 2. Material and Methods

### 2.1. Analyzed Biosensor Configurations

**Figure 1:**
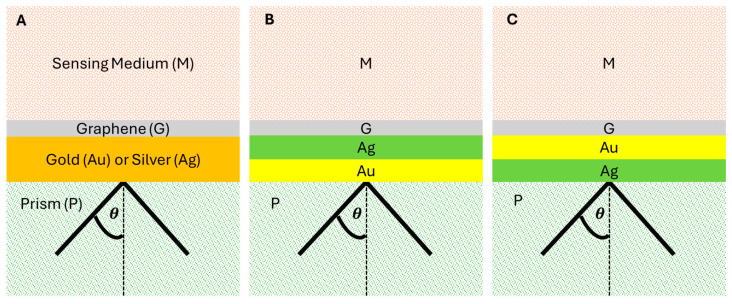
Biosensor illustration. Proposed biosensors by using different metallic substrate configurations: (**A**) gold or silver, (**B**) silver/gold, and (**C**) gold/silver.

The schematic illustration of the proposed SPR biosensors using one- or two-metallic configurations is shown in Figure 1. We point out that the top surface of the SF10 prism accommodates an index-matching SF10 glass slide as for the BK7 prism-glass slide [32]. Under this assumption, these structures contain four (or five) layers based on the Kretschmann configuration [33]:

- Figure 1A: P/Au or Ag/G/M
- Figure 1B: P/Ag/Au/G/M
- Figure 1C: P/Au/Ag/G/M

Here, the SF10 glass prism is chosen for its high refractive index (*n* ≈ 1.7231) [14] (Table 1), which enhances the coupling efficiency of incident light into the sensor. The higher refractive index of SF10 glass compared to other materials like the BK-7 glass prism (*n* ≈ 1.517) [32] allows for better phase matching conditions, essential for efficient SPR excitation. This results in a more pronounced evanescent field, improving the sensitivity of the biosensor. 

We use an SF10 glass prism as the coupling prism, followed by one or two metallic layers made of gold or silver. Gold, known for its chemical stability and excellent plasmonic properties, is a preferred choice for SPR biosensors. Its oxidation resistance ensures longevity and consistent performance. Instead, silver offers superior plasmonic properties, resulting in sharper SPR signals and higher sensitivity. However, gold has a limitation in that biomolecules do not adsorb efficiently onto it, leading to lower detection accuracy and sensitivity. Additionally, the silver layer tends to oxidize when in contact with biomolecules, which reduces the lifetime of the SPR biosensor. To circumvent these problems, recently, it has been proposed to use bimetallic (Ag/Au) configurations to improve the sensitivity of SPR biosensors [7,14]. However, the issues related to the greater degree of complexity in the production of bimetallic structures and the potentially higher susceptibility to oxidation of the bimetallic layer, require exhaustive research to further motivate their use.

Alternatively, the use of graphene offers two meaningful solutions to the challenges faced by traditional and bimetallic configurations, i.e., (i) protection of metallic substrates and (ii) BRE approach:

(i) Graphene is expected to protect the metallic substrate made of gold, silver, or a combination of both. The high chemical stability and impermeability of graphene can serve as a barrier against oxidation and other environmental factors that can degrade the performance of silver and gold substrates [34]. This protective layer could extend the lifespan and reliability of the biosensor, ensuring consistent performance over time.
(ii) Graphene serves as an effective BRE, improving the interaction with target molecules. The high surface area and strong π-stacking interactions of graphene with biomolecules enhance the adsorption efficiency, leading to higher sensitivity and better detection accuracy [14,35]. This characteristic is crucial for detecting low concentrations of analytes, making graphene-enhanced SPR biosensors highly suitable for various applications.

It is worth noting that the synthesis of graphene on gold and silver has been extensively studied due to its potential applications in various technological fields. For instance, a simple method for creating graphene-silver nanoparticle hybrids on highly oriented pyrolytic graphite (HOPG) substrates has been developed, confirming the effective integration of silver with graphene for enhanced properties [36]. Additionally, research on graphene sheets functionalized with gold and silver nanoparticles highlights their utility in sensing and catalytic applications [37]. Chemical vapor deposition (CVD) methods have also been employed to synthesize graphene using gold, silver, and copper substrates [38]. Direct growth techniques have shown the feasibility of producing graphene on Cu structures and extending this method to gold substrates, enhancing surface properties for various applications [39]. Furthermore, the functionalization of graphene with gold and silver nanostructures has proven effective in surface-enhanced Raman spectroscopy (SERS), which is vital for molecular detection [40].

**Table 1:** SPR biosensor configuration. Thickness, refractive indices, and dielectric constants at 633 nm. For comparison purposes, the parameters of graphite are included.

Component	Thickness (nm)	Refractive Index	Ref.
Prism	-	1.7231	[17]
Gold (Au)	50.0	0.1726 + 3.4218 *i*	[17]
Silver (Ag)	55.0	0.0563 + 4.2760 *i*	[7]
Graphene	0.34	2.7611 + 1.6987 *i*	[41]
Graphite	∞	2.8366 + 1.5946 *i*	[42]
Water	-	1.332	[43]

Lastly, in the sensing medium layer, water can be used for simplicity with a refractive index of *n* ≈ 1.332 + Δ*n*, where Δ*n* is varying due to the BRE-analyte interaction (it is assumed after adsorption a Δ*n* = 0.005 [17]). Additionally, graphene could absorb the biomolecules, for instance, ssDNA with a thickness of 100 nm, and produce a local increase in Δ*n* [17]. The complete parameters of scrutinized biosensors are reported in Table 1; for comparison, the parameters of graphite are also reported. 

### 2.2. Theoretical Framework

We have performed a numerical analysis to determine the reflectance of the systems under investigation using TMM and Fresnel questions [7,17,44]. As stated, the proposed biosensors consist of a prism, one metal or two metals, graphene, and a sensing medium (water) being placed in parallel one after another (Figure 1). The thickness of each layer is represented in the perpendicular direction (*z*-axis). The boundary conditions at the interface of the first and last layers are denoted as *Z* = *Z*_1_ = 0 and $Z=Z_{n-1}$, respectively. We point out that the (complex) refractive index (RI) of the different layers is equal to the square root of its (complex) dielectric constant.

The transfer matrix expresses a relationship between the tangential components of electric and magnetic fields of the first and last layers, as follows:

(1) $$\left[\begin{array}{c} E_{1}\\ H_{1} \end{array}\right]=M\left[\begin{array}{c} E_{N-1}\\ H_{N-1} \end{array}\right]$$

Here, E_1_, H_1_, $E_{N-1}$, and $H_{N-1}$ represent the tangential components of electric and magnetic fields at the first and last layer interfaces, respectively. M represents the characteristic matrix of N-layer structure with elements *M_ij_*, as follows:

(2) $$M=\overset{N-1}{\underset{k=2}{\prod }}M_{k}=\left[\begin{array}{cc} M_{11} & M_{12}\\ M_{21} & M_{22} \end{array}\right]$$

And *M_k_* is denoted as:

(3) $$M_{k}=\left[\begin{array}{cc} \cos\beta_{k} & -i\sin\beta_{k}/q_{k}\\ -iq_{k}\;\sin\beta_{k} & \cos\beta_{k} \end{array}\right]$$

where, *k* represents an arbitrary integer number. Additionally, *β_k_* represents the phase thickness and *q_k_* represents the refractive index of the corresponding layer which are expressed as:

(4) $$\beta_{k}=\frac{2\pi d_{k}}{\lambda_{0}}\sqrt{\varepsilon_{k}-n_{1}^{2}\sin^{2}\theta }$$

and

(5) $$q_{k}=\frac{\sqrt{\varepsilon_{k}-n_{1}^{2}\sin^{2}\theta }}{\varepsilon_{k}}$$

where, *θ* is the angle of incidence, $\lambda_{0}$ is the wavelength of the incident light, $n_{1}$ is the refractive index of the prism, *d_k_* is the thickness layer, and the local dielectric function $\varepsilon \left(\lambda_{0}\right)$ can be adopted as $n\left(\lambda_{0}\right)$. For comparison with future experiments, we use the He-Ne laser as a light source with $\lambda_{0}=633$ nm. After straightforward calculations, the total reflection of the N-layer system is:

(6) $$R={\left|\frac{\left(M_{11}+M_{12}q_{N}\right)q_{1}-\left(M_{21}+M_{22}q_{N}\right)}{\left(M_{11}+M_{12}q_{N}\right)q_{1}+\left(M_{21}+M_{22}q_{N}\right)}\right|}^{2}$$

then, by using Equation (6) the SPR curve (i.e., the reflectance as a function of angle of incidence) can be obtained. We point out that for each SPR curve, the excitation of surface plasmons is recognized as a minimum in the reflected intensity *R* (i.e., the attenuated total reflection (ATR) minimum). The angle of incidence at ATR minimum is called the SPR angle. 

### 2.3. Performance Metrics of the SPR Biosensor

The performance metrics of the SPR sensor are characterized by mostly four parameters: sensitivity (*S^L^*), sensitivity to the refractive index change ($S_{RI}^{L}$), detection accuracy (DA), and quality factor (QF).

- The sensitivity of the biosensors (*S^L^*) is defined as the multiplication of the sensitivity to the refractive index change ($S_{RI}^{L}$) and the adsorption efficiency of the target analyte (*E*) as:
  (7) $$S^{L}=S_{RI}^{L}\cdot E$$

In this work, owing to experimental values are needed for *E*, we focus on the sensitivity enhancement concerning conventional biosensors (i.e., without graphene, L0) denoted as:

(8) $$\Delta S_{RI}^{L}=(S_{RI}^{L}-S_{RI}^{0})/S_{RI}^{0}$$

- The sensitivity to the refractive index change can be denoted as:

(9) $$S_{RI}^{L}=\Delta \theta /\Delta n$$

The parameter Δ*θ* represents the angle variation before/after adsorption.

- The detection accuracy (DA) or signal-to-noise ratio can be expressed in terms of angle change (Δ*θ*) and full width at half maximum (FWHM) as:

(10) DA=Δθ/FWHM

- Finally, quality factor (QF) can be expressed in terms of S and FWHM as:

(11) $$\mathrm{QF}=S_{RI}^{L}/\mathrm{FWHM}$$

## 3. Results and Discussion

In this work, we are not focusing on any specific biomolecule targets such as single-stranded DNA (ssDNA), proteins, or virus elements. Our primary interest lies in investigating the fundamental physical properties associated with the interaction of graphene and metal substrates. This approach allows us to explore the intrinsic characteristics of the sensor configurations without being influenced by the particularities of different analytes. Consequently, our study aims to fill the gap in understanding the interaction of graphene with metallic substrates and its impact on the overall performance of SPR biosensors. This focus ensures that our findings can be universally applied to a wide range of biosensing applications, irrespective of the specific target biomolecules, which include but are not limited to RNA, antibodies, and small molecules like glucose. To support this focus, we used a refractive index change: Δn=0.005 for our theoretical analysis, which can be adjusted based on experimental evidence to reflect practical conditions. By emphasizing these physical properties, we avoid the need for comparative work with various analytes, which is beyond the scope of this study.

### 3.1. Reflectance

**Figure 2:**
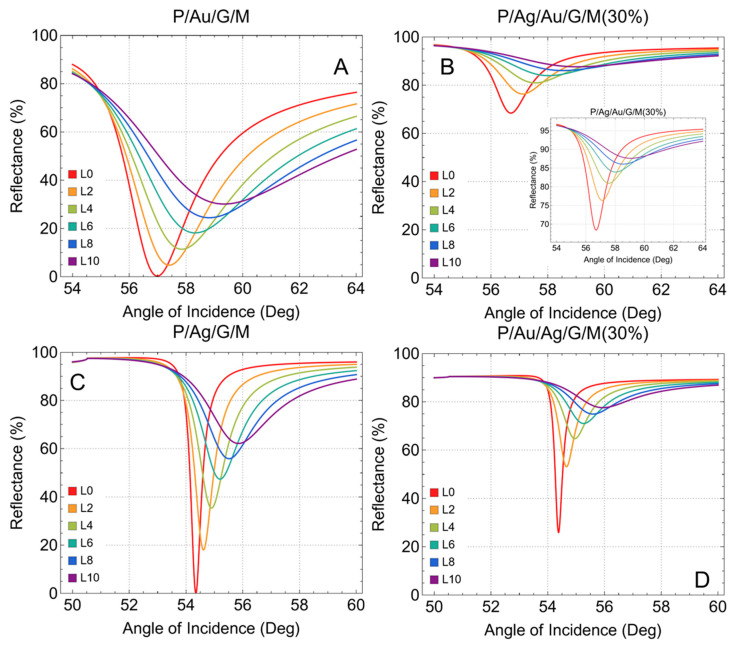
Reflectance (%) before adsorption. SRP curves as a function of the angle of incidence (°), increasing the number of graphene layers from L0 (no graphene layer) to L9 (nine graphene layers). (**A**) prism/gold/graphene/sensing medium, (**B**) prism/silver/gold/graphene/sensing medium, (**C**) prism/silver/graphene/sensing medium, and (**D**) prism/gold/silver/graphene/sensing medium. (**B**,**D**) correspond to the calculations by reducing the thickness of each metallic substrate by 30%.

**Figure 3:**
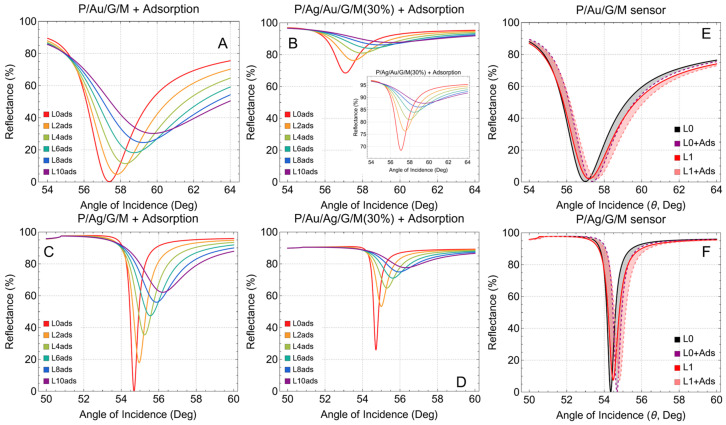
Reflectance (%) after adsorption. SRP curves as a function of the angle of incidence (°), increasing the number of graphene layers from L0 (no graphene layer) to L9 (nine graphene layers). (**A**) prism/gold/graphene/sensing medium, (**B**) prism/silver/gold/graphene/sensing medium, (**C**) prism/silver/graphene/sensing medium, and (**D**) prism/gold/silver/graphene/sensing medium. (**B**,**D**) correspond to the calculations by reducing the thickness of each metallic substrate by 30%. (**F**) and (**G**) SPR resonance curves before/after adsorption for the conventional sensor (L0 and L0 + Ads) and the monolayer graphene sensor (L1 and L1 + Ads), assuming a refractive index change Δ*n* = 0.005, for the P/Au/G/M and P/Ag/G/M sensors, respectively.

Reflectance is a crucial parameter in SPR biosensors as it directly affects the sensitivity and accuracy of detection. We begin analyzing the reflectance curves for various configurations of graphene-based SPR biosensors, both before (Figure 2) and after (Figure 3) adsorption, and with different thicknesses of metallic substrates.

Figure 2A shows the reflectance curves for the P/Au/G/M configuration before adsorption. As the number of graphene layers increases from L0 (no graphene) to L9 (nine graphene layers), the resonance dip becomes wider and shifts slightly. Similarly, Figure 2C shows the reflectance curve for the P/Ag/G/M configuration, where is observed a more pronounced resonance dip compared to gold which can be attributed to the superior plasmonic properties of silver.

Interestingly, Figure S1A shows the reflectance curves for the P/Ag/Au/G/M configuration before adsorption, evidencing that thicker metallic layers attenuate the reflectance (i.e., gold thickness = 50 nm and silver thickness = 55 nm, see Table 1), making them less effective for biosensing. In the same context, Figure S1C before adsorption, the P/Au/Ag/G/M configuration also shows poor reflectance characteristics due to the thicker metallic layers. Based on these results, other systems have been proposed with a 30% reduction in the thickness of the metallic substrates, which should not significantly alter the refractive index. Notably, gold and silver substrates with thicknesses of 25 nm (0.223 + 3.540*i*) [45] and 20 nm (0.072 + 3.932*i*) [46] have been experimentally produced. Therefore, the additional systems to be considered in this study are denoted as:

- P/Ag/Au/G/M(30%)
- P/Au/Ag/G/M(30%)

Thus, in the P/Ag/Au/G/M(30%) configuration (Figure 2B), the thickness of each metallic substrate (gold and silver) is reduced by 30%. The original thicknesses of gold (50 nm) and silver (55 nm) are reduced to 35 nm and 38.5 nm, respectively. This reduction recovers the reflectance characteristics, as seen by the sharper resonance dips. The inset highlights the recovered reflectance, showing how reducing the thickness helps in better light coupling and plasmon resonance. Likewise, in the P/Au/Ag/G/M(30%) configuration (Figure 2D), the reflectance characteristics are evident, with sharper and more defined resonance dips, indicative of better performance for biosensing applications. 

In Figure 3, we now focus on the configurations after adsorption. The reflectance curves for the P/Au/G/M configuration (see Figure 3A,F) show a shift in the resonance angle. This shift is due to the increased local refractive index caused by biomolecule adsorption (Δ*n* = 0.005), further highlighting the enhanced sensitivity with graphene layers. Also, the P/Ag/G/M configuration exhibits a pronounced shift in the resonance angle after adsorption (see Figure 3C,G), indicating high sensitivity. Similarly, the configurations P/Ag/Au/G/M(30%) (Figure 3B) and P/Au/Ag/G/M(30%) (Figure 3D), are characterized by a noticeable shift in the resonance angle and the reduced thickness helps maintain sharp resonance dips even after adsorption, ensuring high sensitivity. The thicker metallic configurations (Figure S1B,D) show similar attenuation, confirming that such configurations are not ideal for high-performance biosensors which reinforces the need for optimized thickness in metallic substrates for effective biosensing. Progress on this idea is constantly growing [47,48,49].

**Figure 4:**
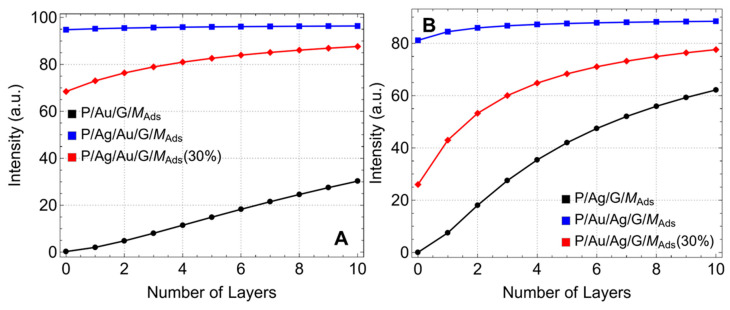
ATR minimum (%). Reflectance intensity as a function of the number of graphene layers, considering different metallic substrate configurations. (**A**) graphene first supported on gold after adsorption and (**B**) graphene first supported on silver after adsorption.

To further expand the observed reflectance minimum change and related implications, these results are reported in Figure 4 for the different configurations and after adsorption. In Figure 4A, the P/Au/G/M_ads_ configuration (black curve) shows that the reflectance minimum starts at nearly 0.24% for L0 and increases to approximately 30.11% for L10. This steady increase suggests that the introduction of graphene layers reduces the coupling efficiency slightly but enhances the overall interaction between light and the sensor surface. For instance, at L5, the reflectance minimum is around 14.79%. For the P/Au/G/M_ads_ (30%) configuration (red curve), the reflectance minimum starts at 68.41% for L0 and increases to 87.62% for L10. This significant increase in baseline reflectance indicates less efficient coupling but higher responsiveness to changes introduced by graphene layers. The P/Ag/Au/G/M_ads_ (blue curve) configuration shows a reflectance minimum that remains at nearly 100% for all layers. This high baseline reflectance confirms that the thicker metallic substrates attenuate the light, preventing effective coupling and SPR generation. Similar results were observed before adsorption.

Otherwise, in the P/Ag/G/M_ads_ configuration (Figure 4B, black curve), the reflectance minimum starts at approximately 0.04% for L0 and increases to around 62.14% for L10. The superior plasmonic properties of silver are evident, enhancing sensitivity significantly with more graphene layers. For the P/Au/Ag/G/M_ads_ (30%) configuration (red curve), the reflectance minimum starts at 25.92% for L0 and increases to 77.56% for L10. In the P/Au/Ag/G/M_ads_ configuration (blue curve), the reflectance minimum starts at approximately 81.13% for L0 and increases to around 88.43% for L10. Similar results are also observed before adsorption.

### 3.2. ATR Angle

**Figure 5:**
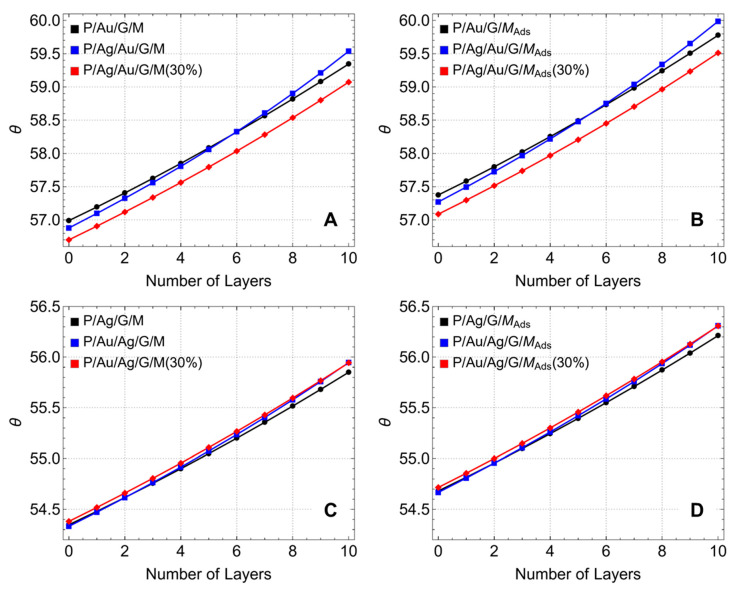
ATR angle (°). Angle position as a function of the number of graphene layers, considering different metallic substrate configurations. (**A**) graphene first supported on gold before adsorption, (**B**) graphene first supported on gold after adsorption, (**C**) graphene first supported on silver before adsorption, and (**D**) graphene first supported on silver after adsorption.

Figure 5 analyzes the position of the reflectance minimum angle (or ATR angle) for different configurations, focusing on how this angle shifts to higher values before/after adsorption as the number of graphene layers increases. In the P/Au/G/M configuration (black curve, before adsorption, Figure 5A), the angle position starts at around 56.99° for L0 and increases to approximately 59.35° for L10 following a quasi-linear trend. For the P/Ag/Au/G/M configuration (blue curve), the angle position starts at around 56.88° for L0 and follows a parabolic trend, reaching approximately 59.54° for L10. As noted, the thicker metallic layers in this configuration attenuate the incident light, resulting in a different trend compared to thinner configurations. Actually, for the P/Ag/Au/G/M(30%) configuration, the angle position starts at around 56.70° for L0 and increases to roughly 59.07° for L10, most importantly, the quasi-linear trend is recovered, suggesting that reducing the thickness of the metallic layers helps maintain the sensitivity and responsiveness of the sensor. After adsorption, the same quasi-linear and parabolic trends are observed in Figure 5B. However, the change towards higher angle values is evident as an effect of adsorption concerning their counterparts.

On the other hand, when graphene is first supported on silver, all configurations exhibit a quasi-linear trend (Figure 5C,D), and the change towards higher angle values is less pronounced. To clarify this fact, in the P/Ag/G/M configuration (black curve, before adsorption, Figure 5C), the angle position starts at around 54.35° for L0 and increases to approximately 55.85° for L10. For the P/Au/Ag/G/M configuration (blue curve), the angle position starts at around 54.33° for L0 reaching approximately 55.95° for L10. Similarly, in the P/Au/Ag/G/M(30%) configuration (red curve), the angle position starts at around 54.38° for L0 and increases to nearly 55.94° for L10. After adsorption (Figure 5D), all configurations display the change towards larger angle values.

To emphasize these findings, the observed shift to higher angle values after adsorption is attributed to the increase in the local refractive index due to the adsorption of biomolecules on the sensor surface. This shift confirms the enhanced sensitivity to changes in the local environment, which is crucial for detecting low concentrations of analytes. Additionally, the parabolic trend observed in configurations with thicker metallic layers is attributed to the attenuation of light. This attenuation affects the coupling efficiency and results in a different angle shift behavior compared to the quasi-linear trend observed in configurations with reduced thickness. Understanding this behavior is crucial to have an idea of what is observed experimentally, which will allow optimizing the sensor design and achieving the desired sensitivity and performance. Lastly, the angle shift is more pronounced when graphene is supported first on gold compared to when it is first supported on silver. This is likely due to the different optical properties of gold and silver, with gold providing better stability and interaction with graphene, leading to a more significant shift in the resonance angle [50]. 

### 3.3. Normalized SPR Curve

**Figure 6:**
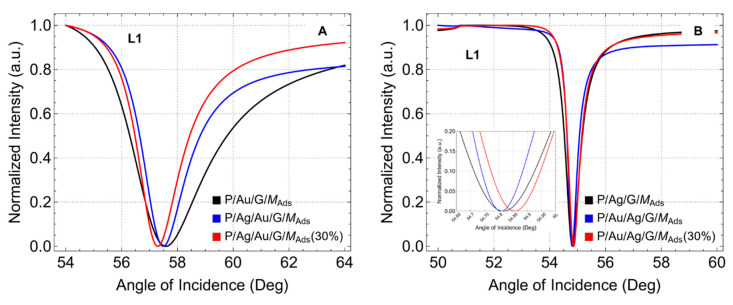
Normalized Reflectance after adsorption. SRP curves as a function of the angle of incidence (°), considering one graphene layer (L1). (**A**) Graphene first supported on gold and (**B**) Graphene first supported on silver.

**Figure 7:**
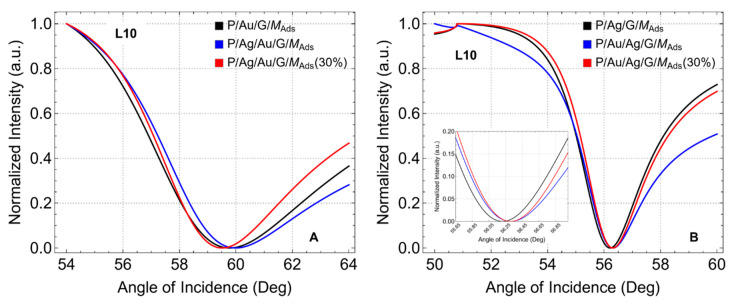
Normalized Reflectance after adsorption. SRP curves as a function of the angle of incidence (°), considering ten graphene layers (L10). (**A**) Graphene first supported on gold and (**B**) Graphene first supported on silver.

To further emphasize the previous findings. We now analyze the normalized reflectance curves for the configurations after adsorption, focusing specifically on configurations with one graphene layer (L1) (Figure 6) and ten graphene layers (L10) (Figure 7). Equivalent results are observed for the configurations before adsorption.

In the P/Au/G/M (black curve), P/Ag/Au/G/M (blue curve), and P/Ag/Au/G/M (30%) (red curve) configurations (Figure 6A), the reflectance minimum angles are found at 57.58°, 57.49°, and 57.30°, respectively. These slight shifts to lower angle values suggest that the introduction of a silver layer beneath the gold modifies the optical properties of the biosensor. The reduced thickness of the metallic layers in the P/Ag/Au/G/M (30%) configuration further shifts the resonance angle. Additionally, the width of the normalized reflectance curve is reduced for the bimetallic configurations, with the P/Ag/Au/G/M (30%) configuration exhibiting the narrowest width. This narrower width indicates higher resolution and better detection accuracy, as the sensor can more precisely detect changes in the refractive index. The reduced width is attributed to the presence of silver.

In contrast, for the P/Ag/G/M (black curve), P/Au/Ag/G/M (blue curve), and P/Au/Ag/G/M (30%) (red curve) configurations (Figure 6B), the reflectance minimum angles are observed at 54.58°, 54.62°, and 54.85°, respectively. These slight shifts to higher angle values indicate that the introduction of a gold layer beneath the silver also alters the optical properties of the biosensor. Additionally, the width of the normalized reflectance curve is reduced for the bimetallic configurations, although this change is less pronounced compared to when graphene is first supported on gold. The presence of silver contributes to the narrower width observed, as confirmed by the data.

For the configurations where graphene is first supported on gold (Figure 7A), the reflectance minimum angles shift to higher values with ten graphene layers (L10) compared to one layer (L1). This shift suggests that increasing the number of graphene layers enhances the plasmonic coupling, altering the optical environment more drastically and leading to a higher resonance angle. Additionally, the width of the resonance curves increases, indicating a broader resonance peak which may result in a trade-off between sensitivity and resolution. In contrast, for the configurations where graphene is first supported on silver (Figure 7B), the reflectance minimum angles remain consistent with the L1 configuration. This finding suggests that the interaction between silver and graphene reaches a saturation point, beyond which additional layers have minimal impact on further shifting the resonance angle. These findings emphasize the importance of optimizing the number of graphene layers to achieve a balance between sensitivity and resolution in SPR biosensor design.

In addition, to expand the body knowledge on these findings, we hypothesize the following ideas. When graphene is first supported on gold, the reflectance minimum angle shifts to lower values (L1) due to the strong plasmonic coupling and superior stability provided by gold, which enhances the interaction with graphene and leads to a resonance shift. The properties of gold, such as its high conductivity and resistance to oxidation, create a stable environment that allows for efficient plasmon propagation, resulting in a lower resonance angle. In contrast, when graphene is first supported on silver (L1 or L10), the reflectance minimum angle shifts to higher values. Silver has superior plasmonic properties but is more susceptible to oxidation and less stable than gold. The introduction of gold beneath the silver layer modifies the optical environment, leading to an increased resonance angle. The difference in behavior can be attributed to the distinct optical interactions and stability characteristics of gold and silver, where gold supports more stable and efficient plasmon resonance at lower angles, while silver, despite its high plasmonic efficiency, leads to higher resonance angles due to its less stable nature and higher susceptibility to environmental changes.

### 3.4. Sensitivity Enhancement

**Figure 8:**
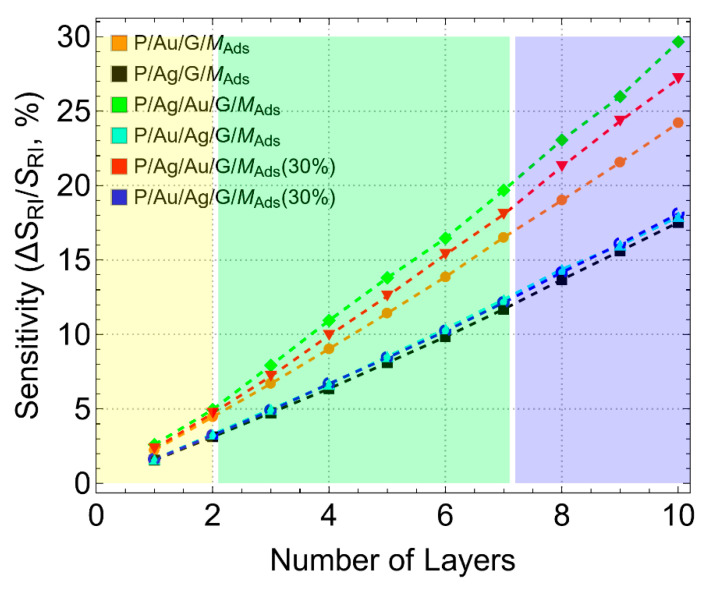
Sensitivity enhancement (%). Sensitivity variation with reference to the conventional biosensor as a function of the number of graphene layers, considering different metallic substrate configurations.

Moving forward, we now concentrate on the performance metrics of the biosensor. To begin, we analyze the sensitivity enhancement of various SPR biosensor configurations concerning the conventional biosensor (without graphene) as a function of the number of graphene layers, ranging from 1 to 10 layers. Figure 8 shows the results obtained using Equation (8), where the angle variation after adsorption for different metallic substrate configurations (as shown in Figure S2) was used. Note that we have added the prefix “ads” to point out obviously that the results are after adsorption.

For configurations where graphene is first supported on gold, a steady increase in sensitivity enhancement is observed. Specifically, the P/Au/G/M configuration (orange line) reaches up to approximately 25% for 10 graphene layers (L10), which is consistent with previous predictions [17]. This indicates strong plasmonic coupling and enhanced sensitivity when graphene is supported on gold. The P/Ag/Au/G/M_ads_(30%) configuration (red line) demonstrates an even higher sensitivity enhancement, reaching up to about 28% for L10. The introduction of a silver layer beneath the gold further increases sensitivity due to improved plasmonic properties. In addition, the reduced thickness of the metallic layers optimizes light coupling and plasmon resonance, resulting in superior sensitivity. The highest sensitivity enhancement is observed in the P/Ag/Au/G/M_ads_ configuration (green line), reaching up to 30% for L10. However, it is crucial to remember the previously discussed issues with this configuration, where the incident light is almost attenuated.

In contrast, configurations where graphene is first supported on silver show a maximum sensitivity enhancement of around 17% for L10. The P/Ag/G/M_ads_ configuration (black line) reaches this level, indicating that the interaction between graphene and silver enhances sensitivity, though less pronounced compared to gold-supported configurations. Similarly, the P/Au/Ag/G/M_ads_ (cyan line) and P/Au/Ag/G/M_ads_(30%) (blue line) configurations also show maximum sensitivity enhancements of about 17% for L10. These results suggest that the addition of a gold layer or the reduction of metallic layer thickness does not significantly change the sensitivity when graphene is supported on silver.

In addition, Figure 8 utilizes different colors to indicate regions based on the methods used to obtain graphene. The yellow region represents single or bilayer graphene, typically obtained through CVD methods which is a bottom-up approach [51]. While CVD produces high-quality graphene, it is generally expensive, and the sensitivity enhancement in this region does not exceed 5%. This cost factor makes single or bilayer graphene less practical for widespread biosensor applications. Otherwise, the green region, covering 3 to 7 graphene layers, is dedicated to few-layer graphene obtained via mechanical [52] or liquid [53] exfoliation methods, both of which are top-down approaches. These methods are more cost-effective compared to CVD. Our predictions indicate that using few-layer graphene can significantly enhance the sensitivity of the biosensor while keeping the costs lower than those associated with single or bilayer graphene. The blue region, covering >8 graphene layers pertain to graphene nanoplatelets [54], which are also obtained through exfoliation methods. These nanoplatelets offer an excellent balance between cost and performance, providing superior sensitivity enhancements compared to fewer layers and CVD-graphene. This makes graphene nanoplatelets particularly advantageous for developing high-performance, cost-effective biosensors.

While the maximum sensitivity enhancement is observed for few-layer graphene or graphene nanoplatelets, there are several limitations to consider. One significant challenge is creating homogeneous dispersions on the metallic substrate, which is essential for achieving consistent sensor performance. Additionally, maintaining a consistent number of graphene layers across the entire biosensor is crucial for reliability. Variations in layer number can lead to uneven sensitivity and reduced overall efficiency. Addressing these limitations is vital for optimizing the practical application of graphene-based biosensors and fully realizing their potential benefits.

### 3.5. Full Width Half Maximum (FWHM)

FWHM is a crucial parameter because it appears in Equations (10) and (11) to calculate the detection accuracy and quality factor. Additionally, while sensitivity can be improved, the resolution could be compromised, motivating a proper analysis of FWHM. To remark, the FWHM is a measure of the bandwidth of the resonance peak, and a higher FWHM indicates broader resonance, which can lead to reduced accuracy in detection (sensing) applications. Therefore, accurately assessing the FWHM for various configurations allows for better performance optimization of the biosensor, ensuring that the quality factor and detection accuracy are maximized. Then, we shift our focus to a detailed analysis of the performance metrics for biosensor configurations involving both single metal layers and bimetallic layers with a 30% reduction in thickness. 

**Figure 9:**
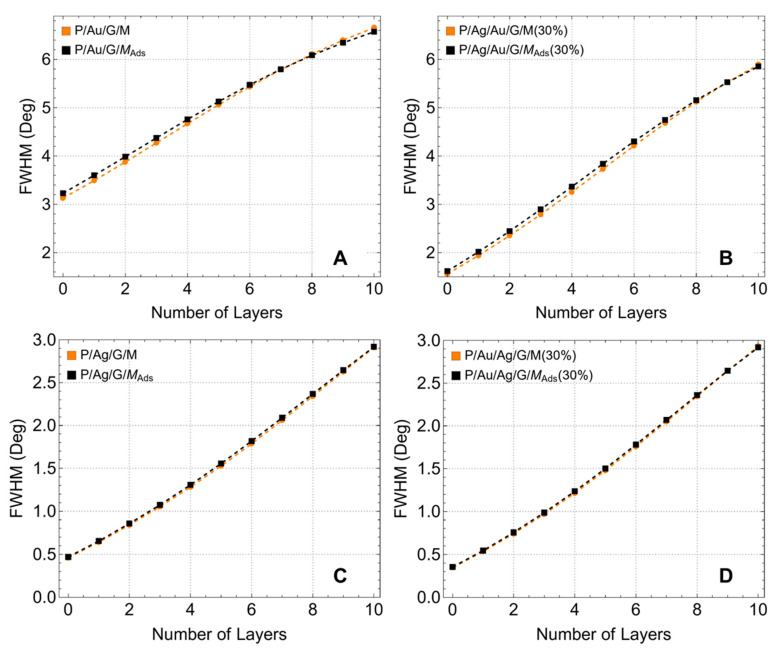
Full width at half maximum (FWHM) analysis (°). FWHM as a function of the number of graphene layers, considering different metallic substrate configurations. (**A**,**B**) graphene first supported on gold before/after adsorption, (**C**,**D**) graphene first supported on silver before/after adsorption. Note that it only included systems with a 30% reduction in each metallic substrate.

Before (orange lines) and after (black lines) adsorption, for configurations where graphene is first supported on gold (Figure 9A,B), the FWHM shows a nearly linear increase with the number of graphene layers, reaching around 6.8° for 10 layers. This trend suggests that the addition of graphene layers steadily increases the resonance width, indicating a clear linear trend up to 6 layers, and from this point, saturation is observed. The minimal change in FWHM before and after adsorption indicates that the adsorption process has little effect on the resonance width for configurations with gold as the initial support. However, it is important to note that in a tinner bimetallic configuration (Figure 9B), the FWHM substantially decreases compared to when using a single metallic layer (Figure 9A).

In contrast, for configurations where graphene is first supported on silver (Figure 9C,D), the FWHM exhibits a clear parabolic trend, increasing from about 0.5° to around 3.0° as the number of graphene layers increases. This parabolic increase suggests that the addition of graphene layers more significantly affects the resonance width when silver is the initial support layer. Unlike the gold-supported configurations, the change in FWHM due to adsorption is negligible, indicating that the adsorption process does not impact the resonance width in silver-supported configurations. The stronger dependency of resonance trend on the number of graphene layers in silver-supported configurations can be attributed to the higher plasmonic efficiency of silver and its susceptibility to environmental changes.

A similar analysis was conducted for the angle change (Δ*θ*), which is a fundamental parameter in Equations (9)–(11) to obtain the sensitivity to the refractive index change and detection accuracy. The observed results are presented in Figure S2, with numerical values detailed in Tables S1–S4. Small variations of less than 0.45° were observed, which is a direct consequence of the Δ*n* used in this study. It is expected that larger Δ*n* values would result in more significant Δ*θ* changes [7].

### 3.6. Effect on Sensitivity, Detection Accuracy, and Quality Factor

**Figure 10:**
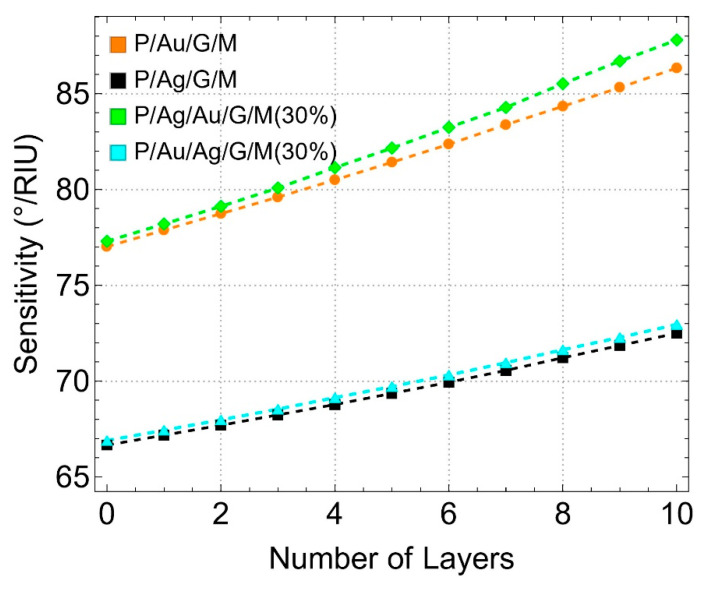
Sensitivity to refractive index change. Sensitivity (°/*RIU*) as a function of the number of graphene layers, considering different metallic substrate configurations. Note that it only included systems with a 30% reduction in each metallic substrate.

**Figure 11:**
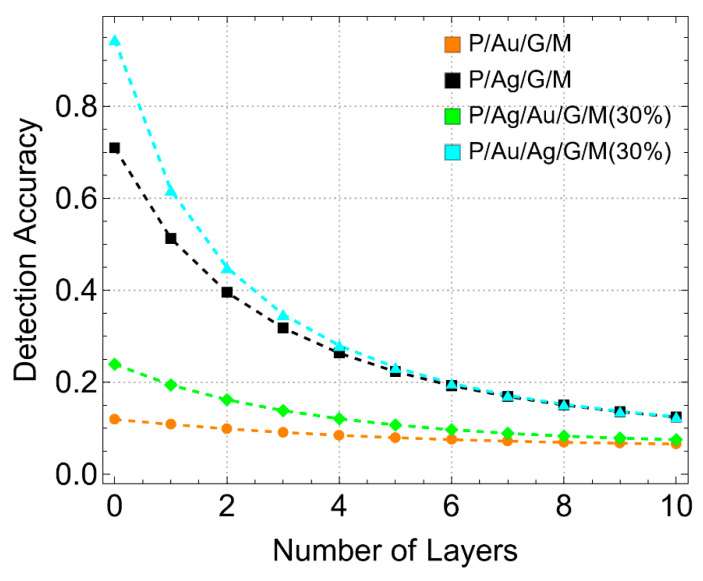
Detection Accuracy (DA). DA (dimensionless) as a function of the number of graphene layers, considering different metallic substrate configurations. Note that it only included systems with a 30% reduction in each metallic substrate.

**Figure 12:**
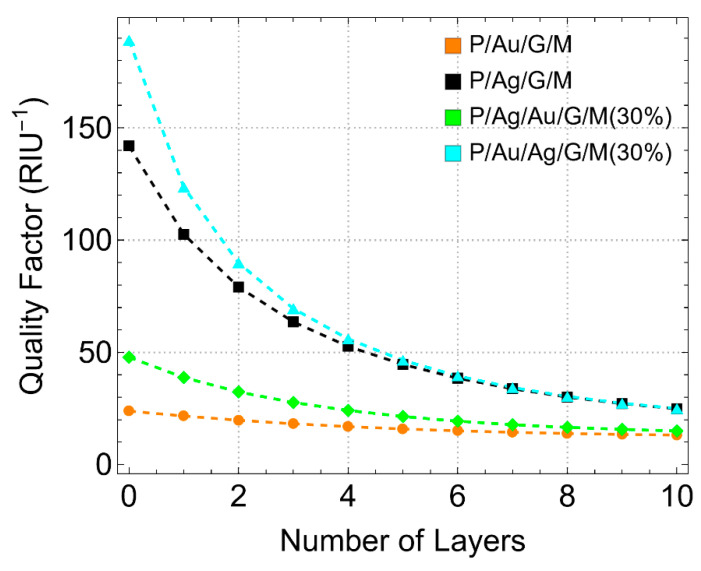
Quality Factor (QF). QF (*RIU*^−1^) as a function of the number of graphene layers, considering different metallic substrate configurations. Note that it only included systems with a 30% reduction in each metallic substrate.

In this subsection, we analyze the effect of the number of graphene layers on the sensitivity to refractive index change (Figure 10), detection accuracy (Figure 11), and quality factor (Figure 12) of the biosensor. The respective numerical values are reported in Tables S1–S4.

Figure 10 shows the sensitivity (°/RIU) as a function of the number of graphene layers for different configurations. As observed, the sensitivity increases linearly with the number of graphene layers for all configurations. Specifically, the P/Au/G/M configuration (orange line) reaches approximately 86.34°/RIU for 10 graphene layers (L10), demonstrating strong plasmonic coupling and enhanced sensitivity due to the interaction between graphene and gold. The P/Ag/Au/G/M (30%) configuration (green line) exhibits the highest sensitivity, reaching about 87.80°/RIU for L10. This indicates that the introduction of a silver layer beneath the gold and the reduced thickness of the metallic layers optimize light coupling and plasmon resonance, resulting in superior sensitivity. In contrast, the P/Ag/G/M configuration (black line) shows lower sensitivity, around 72.50°/RIU for L10, suggesting that silver provides good sensitivity but is less pronounced compared to gold-supported configurations. These results highlight the importance of the substrate material and its thickness in achieving high sensitivity in SPR biosensors. For the P/Ag/Au/G/M (30%) configuration (cyan color), a maximum sensitivity of about 72.96°/RIU for L10 is noted.

Figure 11 shows the detection accuracy (DA) results as a function of the number of graphene layers. DA is inversely related to the FWHM, meaning a narrower resonance width results in higher detection accuracy. For configurations where graphene is first supported on gold (P/Au/G/M and P/Ag/Au/G/M (30%)), DA decreases slightly with the number of graphene layers, starting from about 0.2 and stabilizing around 0.1. This minimal change suggests that gold-supported configurations maintain consistent performance with increasing graphene layers. In contrast, configurations, where graphene is first supported on silver (P/Ag/G/M and P/Au/Ag/G/M (30%)), show a more pronounced decrease in DA, from approximately 0.9 to around 0.2 for L10. The trend observed in these configurations indicates a stronger dependency of DA on the number of graphene layers. These findings emphasize the need to balance sensitivity and detection accuracy, especially in silver-supported configurations.

Figure 12 presents the quality factor (QF) as a function of the number of graphene layers. QF is a critical metric for assessing the overall performance of SPR biosensors, combining both sensitivity and FWHM. Configurations with graphene first supported on gold (P/Au/G/M and P/Ag/Au/G/M (30%)) show a slight decrease in QF with the number of graphene layers, indicating better performance. For example, the P/Ag/Au/G/M (30%) configuration achieves a QF of around 15.0 RIU^−1^ for L10. Conversely, configurations with graphene first supported on silver (P/Ag/G/M and P/Au/Ag/G/M (30%)) exhibit a critical decrease in QF, reaching approximately 25 RIU^−1^ for L10. This pronounced decrease in QF indicates that while silver enhances plasmonic efficiency, it also introduces challenges related to stability and environmental susceptibility.

## 4. Conclusions

In summary, we conducted a comprehensive theoretical analysis of graphene-based SPR biosensors, focusing on various configurations with single and bimetallic layers. Our goal was to understand the effect of different metallic substrates and the number of graphene layers on the sensitivity of detection, DA, and QF. We specifically analyzed the configurations where graphene is supported on gold or silver, and by considering configurations with a 30% reduction in the thickness of the metallic layers to optimize performance and recover the properties of the biosensor.

In particular, the sensitivity to refractive index changes increased with the number of graphene layers across all configurations. The P/Au/G/M configuration showed a sensitivity of detection enhancement of up to approximately 25% for 10 graphene layers (L10), while the P/Ag/Au/G/M configuration exhibited the highest sensitivity of detection, reaching about 30% for L10. In contrast, the P/Ag/G/M configuration demonstrated a maximum sensitivity of detection enhancement of around 17% for L10. The DA varied significantly between gold and silver-supported configurations. Gold-supported configurations maintained a nearly stable DA around 0.1–0.2 with increasing graphene layers, indicating consistent performance. Conversely, silver-supported configurations showed a more pronounced decrease in DA from approximately 0.9 to 0.2 for L10, reflecting the stronger dependency of DA on the number of graphene layers. The QF shows a slight decrease by increasing the number of graphene layers for gold-supported configurations. The P/Ag/Au/G/M (30%) configuration achieved a QF of around 15.0 RIU^−1^. Silver-supported configurations showed a more pronounced decrease in QF, reaching approximately 25.0 RIU^−1^ for L10, indicating challenges related to stability and environmental susceptibility despite enhanced plasmonic efficiency.

In addition, configurations where graphene is first supported on gold demonstrated superior performance metrics, including higher sensitivity of detection, stable DA, and a slight affected in QF. These findings highlight the suitability of gold as a substrate for high-performance SPR biosensors. In this context, reducing the thickness of the metallic layers by 30% in bimetallic configurations optimized light coupling and enhanced sensor performance. The P/Ag/Au/G/M (30%) configuration, in particular, showed the best overall performance, balancing sensitivity of detection, detection accuracy, and quality factor. Future studies should explore the effects of varying metallic layer thicknesses beyond the 30% reduction to identify the optimal configuration for different sensing applications. Furthermore, given the high plasmonic efficiency but greater environmental susceptibility of silver, further research should focus on improving the stability of silver-supported configurations, possibly through protective coatings or hybrid materials.

Finally, while our study provides a theoretical basis for the performance of graphene-based SPR biosensors, experimental validation is crucial to confirm these findings and address practical challenges in biosensor fabrication and deployment. The insights gained from this study pave the way for developing high-performance, reliable SPR biosensors for a wide range of biosensing applications.

## Data Availability

The original contributions presented in the study are included in the article/Supplementary Materials, further inquiries can be directed to the corresponding author.

## Supplementary Materials

The following supporting information can be downloaded at: https://www.mdpi.com/article/10.3390/s24155049/s1. Figure S1. Reflectance (%) before and after adsorption. SRP curves as a function of the angle of incidence (°), increasing the number of graphene layers from L0 (no graphene layer) to L9 (nine graphene layers). (A) prism/silver/gold/graphene/sensing medium, (B) prism/silver/gold/graphene/sensing medium, (C) prism/gold/silver/graphene/sensing medium, and (D) prism/gold/silver/graphene/sensing medium. Figure S2. Angle variation (∆θ). Angles change as a function of the number of graphene layers, considering different metallic substrate configurations. Note that it only included systems with a 30% reduction in each metallic substrate. The angle variation is obtained by the results presented in Figure 5 (main text). Table S1. The performance parameters of the proposed biosensor: prism/gold/graphene/sensing medium. Table S2. The performance parameters of the proposed biosensor: prism/silver/graphene/sensing medium. Table S3. The performance parameters of the proposed biosensor: prism/silver/gold/graphene/sensing medium. Table S4. The performance parameters of the proposed biosensor: prism/gold/silver/graphene/sensing medium.
