# Supplementary material for: The Tunable Parameters of Graphene-Based Biosensors
Source: Sensors (Basel). 2024 Aug 4;24(15):5049. doi: 10.3390/s24155049 (PMC11314989; doi:10.3390/s24155049)
Supplement: Supplementary file 1 [file sensors-24-05049-s001.zip › sensors-3108758-supplementary.pdf]

Supplementary Information: The Tunable Parameters of Graphene-Based Biosensors

Talia Tene[1], Jiří Svozilík[2], Dennys Colcha[3], Yesenia Cevallos[4,5], Paola G. Vinueza-Naranjo[4], Cristian Vacacela Gomez[6,*], Stefano Bellucci[6,*]

[1]Department of Chemistry, Universidad Técnica Particular de Loja, 110160 Loja, Ecuador
[2]Facultad de Ciencias, Escuela Superior Politécnica de Chimborazo (ESPOCH), Riobamba 060155, Ecuador
[3]UNICARIBE Research Center, University of Calabria, 87036 Rende, Italy
[4]College of Engineering, Universidad Nacional de Chimborazo, 060108 Riobamba, Ecuador
[5]Universidad San Francisco de Quito IMNE, Diego de Robles s/n Cumbayá Quito, Ecuador
[6]INFN-Laboratori Nazionali di Frascati, Via E. Fermi 54, I-00044 Frascati, Italy

*Correspondence: vacacela@lnf.infn.it (C.V.G); bellucci@lnf.infn.it (S.B.)

Supplementary Figures

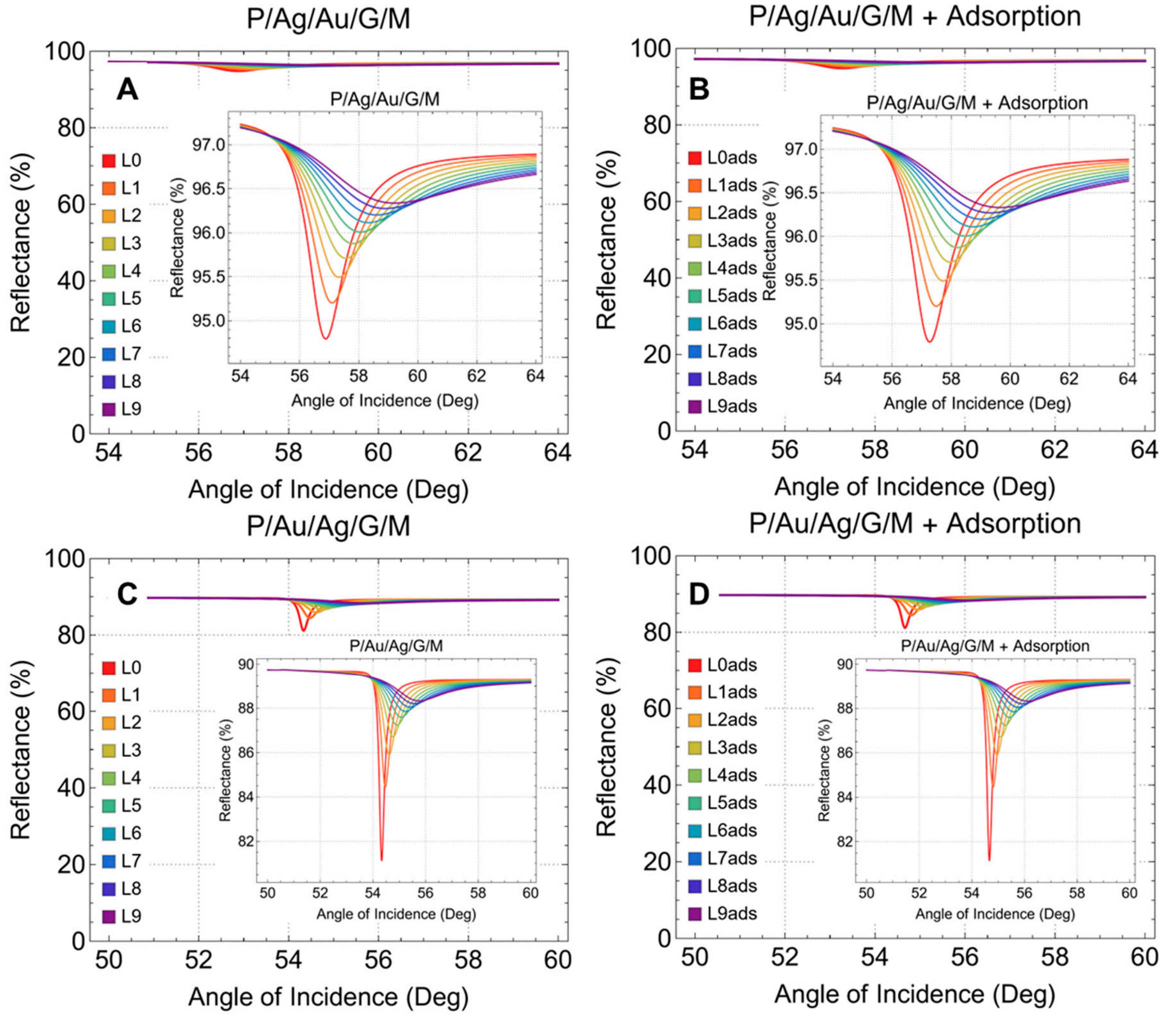

Figure S1. Reflectance (%) before and after adsorption. SRP curves as a function of the angle of incidence (°), increasing the number of graphene layers from L0 (no graphene layer) to L9 (nine graphene layers). (A) prism/silver/gold/graphene/sensing medium, (B) prism/silver/gold/graphene/sensing medium, (C) prism/gold/silver/graphene/sensing medium, and (D) prism/gold/silver/graphene/sensing medium.

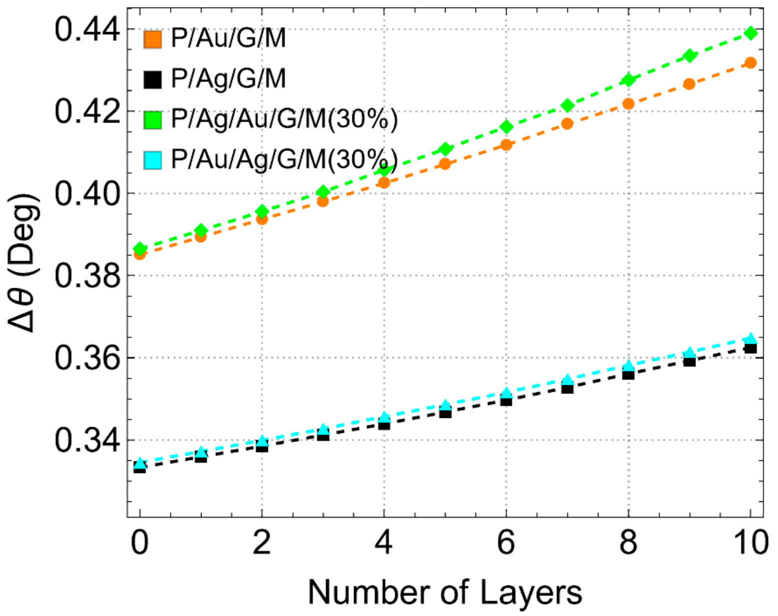

Figure S2. Angle variation ($\Delta\theta$). Angles change as a function of the number of graphene layers, considering different metallic substrate configurations. Note that it only included systems with a 30% reduction in each metallic substrate. The angle variation is obtained by the results presented in Figure 5 (main text).

Supplementary Tables

Table S1. The performance parameters of the proposed biosensor: prism/gold/graphene/sensing medium.

**Prisma / Gold / Graphene / Sensing Medium (P/Au/G/M)**					
No. Layer	$\Delta\theta$ (°)	FWHM	S ($°/RIU$)	DA	QF (RIU^{-1})
0	0.385	3.228	77.02	0.119	23.858
1	0.389	3.599	77.88	0.108	21.642
2	0.394	3.984	78.74	0.099	19.766
3	0.398	4.373	79.60	0.091	18.204
4	0.403	4.756	80.50	0.085	16.926
5	0.407	5.125	81.42	0.079	15.887
6	0.412	5.472	82.36	0.075	15.051
7	0.417	5.793	83.38	0.072	14.394
8	0.422	6.083	84.34	0.069	13.865
9	0.427	6.342	85.32	0.067	13.453
10	0.432	6.571	86.34	0.066	13.140

Table S2. The performance parameters of the proposed biosensor: prism/silver/graphene/sensing medium.

**Prisma / Silver / Graphene / Sensing Medium (P/Ag/G/M)**					
No. Layer	$\Delta\theta$ (°)	FWHM	S ($°/RIU$)	DA	QF (RIU^{-1})
0	0.333	0.470	66.67	0.710	141.900
1	0.336	0.655	67.18	0.513	102.563
2	0.339	0.856	67.70	0.395	79.065
3	0.341	1.074	68.24	0.318	63.571
4	0.344	1.307	68.78	0.263	52.641
5	0.347	1.555	69.36	0.223	44.606
6	0.350	1.817	69.94	0.193	38.497
7	0.353	2.089	70.56	0.169	33.777
8	0.356	2.367	71.22	0.150	30.088
9	0.359	2.646	71.86	0.136	27.163
10	0.363	2.918	72.50	0.124	24.843

Table S3. The performance parameters of the proposed biosensor: prism/silver/gold/graphene/sensing medium.

**Prisma / Silver / Gold / Graphene / Sensing Medium (P/Ag/Au/G/M)**					
No. Layer	$\Delta\theta$ (°)	FWHM	S ($°/RIU$)	DA	QF (RIU^{-1})
0	0.387	1.616	77.30	0.239	47.831
1	0.391	2.015	78.20	0.194	38.804
2	0.396	2.443	79.12	0.162	32.391
3	0.400	2.895	80.08	0.138	27.667
4	0.406	3.363	81.14	0.121	24.128
5	0.411	3.836	82.16	0.107	21.417
6	0.416	4.301	83.24	0.097	19.355
7	0.421	4.743	84.28	0.089	17.768
8	0.428	5.153	85.52	0.083	16.595
9	0.434	5.524	86.70	0.079	15.695
10	0.439	5.852	87.80	0.075	15.003

Table S4. The performance parameters of the proposed biosensor: prism/gold/silver/graphene/sensing medium.

**Prisma / Gold / Silver / Graphene / Sensing Medium (P/Au/Ag/G/M)**					
No. Layer	$\Delta\theta$ (°)	FWHM	S ($°/RIU$)	DA	QF (RIU^{-1})
0	0.335	0.354	66.90	0.945	188.983
1	0.337	0.546	67.44	0.618	123.527
2	0.340	0.757	67.98	0.450	89.836
3	0.343	0.987	68.54	0.347	69.466
4	0.346	1.235	69.14	0.280	55.966
5	0.349	1.501	69.72	0.232	46.444
6	0.352	1.780	70.32	0.198	39.492
7	0.355	2.069	70.96	0.172	34.302
8	0.358	2.359	71.64	0.152	30.369
9	0.361	2.644	72.28	0.137	27.334
10	0.365	2.918	72.96	0.125	25.002
